# Clonal spread of carbapenem-resistant *Klebsiella pneumoniae* among patients at admission and discharge at a Vietnamese neonatal intensive care unit

**DOI:** 10.1186/s13756-021-01033-3

**Published:** 2021-11-20

**Authors:** Björn Berglund, Ngoc Thi Bich Hoang, Ludwig Lundberg, Ngai Kien Le, Maria Tärnberg, Maud Nilsson, Elin Bornefall, Dung Thi Khanh Khu, Jenny Welander, Hai Thanh Le, Linus Olson, Tran Minh Dien, Lennart E. Nilsson, Mattias Larsson, Håkan Hanberger

**Affiliations:** 1 Department of Biomedical and Clinical Sciences, Linköping University, Linköping, Sweden; 2 Vietnam National Children’s Hospital, Hanoi, Vietnam; 3 Department of Molecular Sciences, Swedish University of Agricultural Sciences, Uppsala, Sweden; 4 Training and Research Academic Collaboration (TRAC) – Sweden – Vietnam, Hanoi, Vietnam; 5 Department of Clinical Microbiology and Department of Clinical and Experimental Medicine, Linköping University, Linköping, Sweden; 6 Department of Global Public Health, Karolinska Institutet, Stockholm, Sweden; 7 Department of Women and Children’s Health, Karolinska Institutet, Stockholm, Sweden

**Keywords:** *Klebsiella pneumoniae*, Carbapenem resistance, Carbapenemase, Hospital-acquired infection, Colonisation, Vietnam, Next-generation sequencing

## Abstract

**Background:**

The increasing prevalence of carbapenem-resistant Enterobacteriaceae (CRE) is a growing problem globally, particularly in low- to middle-income countries (LMICs). Previous studies have shown high rates of CRE colonisation among patients at hospitals in LMICs, with increased risk of hospital-acquired infections.

**Methods:**

We isolated carbapenem-resistant *Klebsiella pneumoniae* (CRKP) from faecal samples collected in 2017 from patients at admission and discharge at a Vietnamese neonatal intensive care unit (NICU). 126 CRKP were whole-genome sequenced. The phylogenetic relationship between the isolates and between clinical CRKP isolates collected in 2012–2018 at the same hospital were investigated.

**Results:**

NDM-type carbapenemase-(61%) and KPC-2-encoding genes (41%) were the most common carbapenem resistance genes observed among the admission and discharge isolates. Most isolates (56%) belonged to three distinct clonal clusters of ST15, carrying *bla*_KPC-2_, *bla*_NDM-1_ and *bla*_NDM-4_, respectively. Each cluster also comprised clinical isolates from blood collected at the study hospital. The most dominant ST15 clone was shown to be related to isolates collected from the same hospital as far back as in 2012.

**Conclusions:**

Highly resistant CRKP were found colonising admission and discharge patients at a Vietnamese NICU, emphasising the importance of continued monitoring. Whole-genome sequencing revealed a population of CRKP consisting mostly of ST15 isolates in three clonally related clusters, each related to blood isolates collected from the same hospital. Furthermore, clinical isolates collected from previous years (dating back to 2012) were shown to likely be clonally descended from ST15 isolates in the largest cluster, suggesting a successful hospital strain which can colonise inpatients.

**Supplementary Information:**

The online version contains supplementary material available at 10.1186/s13756-021-01033-3.

## Background

The prevalence of antibiotic-resistant bacteria is increasing globally, leading to elevated healthcare costs and patient mortality. The impact of antibiotic resistance is particularly heavy in low- and middle-income countries (LMICs) [1, 2]. Lower health indicators, higher rates of infection and inability to afford adequate treatment contribute to an elevated burden of infections caused by antibiotic-resistant bacteria in these settings. Furthermore, emergence and dissemination of antibiotic resistance is facilitated by unregulated access to antibiotics and a lack of resources to undertake extensive infection control measures [3]. Data on the burden of carbapenem-resistant Enterobacteriaceae (CRE) is currently scarce in LMICs, however, Stewardson et al. [4] showed in a recent study, with data from ten LMICs, that bloodstream infections caused by CRE were associated with increased length of hospital stays and mortality. In Vietnam, prevalence of hospital-acquired infections (HAIs) at intensive care units (ICUs) has previously been reported to be high. A point prevalence study conducted in 2012 and 2013 found a total prevalence of 30%, with *Klebsiella pneumoniae* being the most common Enterobacteriaceae to cause HAIs, 15% of which were resistant to carbapenems [5]. Carbapenems were the third most prescribed antibiotics, constituting 14% of total antibiotic prescriptions. At the ICUs of three Vietnamese paediatric referral hospitals during the same period, the rate of HAI was reported to be 33% with *K. pneumoniae* being the most common causative agent [6]. 55% of *K. pneumoniae* isolates were reported to be resistant to carbapenems.

Colonisation of the intestinal tract with CRE has been shown to increase the risk of subsequent infection with CRE and to be associated with a higher mortality rate [7, 8]. In a recent study, we reported that 52% of patients admitted to 12 study hospitals in Vietnam were colonised with CRE, most commonly of *K. pneumoniae* (69%) [9]. Risk factors for CRE colonisation included diagnosis with HAI and treatment with a carbapenem. The length of hospital stay was also found to be an independent risk factor. The mean colonisation rate increased from 13% on admission to 89% at day 15. A sub-study at one of the included neonatal ICUs (NICUs) showed ratios of colonisation of 32% at admission and 87% at discharge; a significantly higher mortality rate was observed among patients colonised with CRE. A study among neonatal children with HAIs caused by multidrug-resistant bacteria showed a significant correlation between antibiotic resistance and mortality [10]. These findings prompted further investigation into carriage of carbapenem-resistant *K. pneumoniae* (CRKP) among the patients. In the current study, whole-genome sequencing (WGS) was used to characterise the CRKP colonising the intestinal tract of patients at the abovementioned NICU and to infer the phylogenetic relationships between strains colonising the patients at admission contra discharge. Isolates of CRKP from clinical samples were also characterised to compare the colonising bacteria to strains causing infections at the hospital.

## Materials and methods

### Collection of faecal samples, clinical isolates and patient baseline data

The samples for this study were prospectively collected from patients at a NICU at the largest paediatric hospital in northern Vietnam. The sampling procedure and collection of baseline patient data has been described elsewhere [9]. In brief, between March and June 2017, faecal samples were collected from 326 neonates at admission to the NICU. Because the study hospital does not have a childbirth unit, a few of the admissions are from other wards with the majority of admissions being from other hospitals. Faecal swabs were collected for 248 of these patients at discharge from the NICU. The samples were collected with rectal swabs by using ESwabs (Copan, Brescia, Italy). All patients admitted to the NICU during the study period were included except for patients with anal atresia or other conditions affecting the feasibility of collecting the faecal sample or for whom a caretaker, legal guardian or relative chose to opt out of the study. Patient data collected included sex, date of birth, source of admission, reason for admission, diagnosis, and patient outcome. To compare the findings of CRKP in the faecal samples with the epidemiological situation at the hospital, clinical isolates of CRKP identified in the clinical routine work were retrospectively included in the study. These were constituted of 28 isolates collected from blood from 28 patients in 2017 and 2018, and 13 isolates collected from tracheal aspirates from 12 patients in 2017.

### Screening for CRKP and antimicrobial susceptibility testing

The ESwabs were plated on CarbaID agar (bioMérieux, Marcy l’Étoile, France) and incubated at 35 ˚C for 16 to 24 h. Based on growth on the selective medium and morphology, suspected CRKP were species-determined by using a VITEK 2 (bioMérieux). Isolates determined as CRKP were recultivated on UTI Brilliance agar (Oxoid, Basingstoke, UK). Isolates were also cultured on COL-APSE agar (CHROMagar, Paris, France) to screen for colistin resistance. CRKP originating from the admission (n = 34) and discharge (n = 92) faecal samples, in addition to clinical isolates, were subjected to antimicrobial susceptibility testing to a panel of antibiotics consisting of meropenem, amikacin, gentamicin, tobramycin, cefotaxime, ciprofloxacin, fosfomycin, tigecycline, trimethoprim-sulfamethoxazole, chloramphenicol, tetracycline, ceftazidime-avibactam and colistin. Disc diffusion (Oxoid) on Mueller–Hinton agar plates (Oxoid) was used to test susceptibility to all antibiotics except for colistin, for which broth microdilution with a cation-adjusted Mueller–Hinton broth (BD, Franklin Lakes, NJ, USA) in custom made plates from Sensititre (TrekDS/Thermo Fisher Scientific, Oakwood Village, OH) was used. All zone diameters and MICs were interpreted according to the clinical breakpoints suggested by EUCAST [11] except for tetracycline, for which CLSI guidelines were used [12] and tigecycline. A wild type distribution of tigecycline zone diameters was defined from data from clinical isolates of extended-spectrum β-lactamase-(ESBL)-producing *K. pneumoniae* from Sweden, and isolates were considered resistant if zone diameters were outside of this distribution (i.e., < 15 mm). Multidrug resistance (MDR) was defined as non-susceptibility to antibiotics of three or more antimicrobial categories [13].

### Whole-genome sequencing of CRKP, genome assembly and bioinformatic analysis

Total DNA from 149 CRKP isolates from admission and discharge faecal samples, blood and tracheal aspirates was extracted by using the EZ1 DNA Tissue Kit and the EZ1 Advanced XL instrument (Qiagen, Hilden, Germany) and subsequently stored in – 80 °C. The DNA concentration was determined by using a Qubit 2.0 Fluorometer (Thermo Fisher Scientific, Waltham, MA), and 20 ng of DNA was used as input to construct a sequencing library for whole-genome sequencing. The library preparation was performed by using a QIAseq FX DNA Library Kit (Qiagen) following the manufacturer’s instructions. DNA quantity and quality of the sequencing library was assessed by using a Qubit 2.0 Fluorometer and a QIAxcel instrument (Qiagen), followed by paired-end sequencing performed on a MiSeq instrument (Illumina, San Diego, CA). The raw reads generated were uploaded to the Sequence Read Archive at NCBI (accession numbers: SRR13256598-SRR13246708). An additional 18 isolates collected from admission and discharge faecal samples previously sequenced by using the same methods (accession numbers: SRR12149859-SRR12149874) were also analysed. Genome assembly of the whole-genome sequenced isolates was performed by using CLC Genomics Workbench v.9.5.3 (Qiagen). Multilocus sequence typing (MLST) and querying of antibiotic resistance genes were performed via the databases at the Center for Genomic Epidemiology (http://www.genomicepidemiology.org). Single nucleotide polymorphisms (SNPs) were called against a reference genome (NC_022566) if fulfilling the criteria of having a sequencing depth ≥ 10x, a frequency of ≥ 90% and a Phred score ≥ 20. The resulting variants were used to construct a phylogeny of the sequenced isolates by using CLC Genomics Workbench V.9.5.3. To compare the genetic relatedness of the study isolates to CRKP isolated prior to 2017 at the study hospital from clinical specimen, 5 isolates from 2015 previously reported on elsewhere [14], 4 isolates from 2014, 9 isolates from 2013 and 1 isolate from 2012, all belonging to ST15, were included in the analysis. Isolates differing by ≤ 18 SNPs were considered to constitute a single clone [15]. All raw reads generated and presented in this study are available at the Sequence Read Archive at NCBI (accession numbers: SRR13256598-SRR13246708).

### Statistical analysis

The associations between patient baseline variables sex, treatment outcome and diagnoses with carriage of CRKP were tested with Fisher’s exact tests and χ^2^-tests. Associations between age at admission and length of stay at the NICU with carriage of CRKP were tested with Mann–Whitney U and Kruskal–Wallis one-way analysis of variance tests. Differences in antibiotic susceptibility were assessed with Fisher’s exact tests. To compensate for multiple comparison errors, *P* ≤ 0.01 was considered significant. Due to the low number of patients which were positive at admission but negative at discharge (n = 5), this cohort was excluded from the statistical analysis. All statistical tests were performed in Prism for Windows 8.4.3 and SPSS Statistics version 26.

## Results

### Prevalence of CRKP at admission and discharge and baseline patient statistics

Faecal samples were collected at admission and discharge from a total of 326 patients at the study NICU (Table 1). A total of 69 patients (21%) had faecal samples positive for CRKP at admission and 184 had faecal samples positive for CRKP at discharge (74%). Only one patient which had a faecal sample positive for CRKP at admission had a negative faecal sample at discharge (2.0%). 137 patients (55%) were negative for CRKP at admission but were positive at discharge (i.e., were colonized during their stay at the NICU). A total of 59 patients (24%) had faecal samples which neither tested positive for CRKP at admission nor at discharge.

**Table 1:** Baseline information on patients (n = 326) included in the study on sex, age at admission, length of stay at the neonatal intensive care unit (NICU), treatment outcome and diagnoses

	Patients with admission samples (n = 326)				Patients with both admission and discharge samples (n = 248)										
	Total	Admission +	Admission −	*p*	Total	Discharge +	Discharge −	*p*	Admission + Discharge −	Admission − Discharge +	*p*	Admission − Discharge −	*p*	Admission + Discharge +	*p*
Number of patients	326	69	257	–	248	184	64	–	5	137		59		47	–
*Sex*															
Female	118 (37%)	28 (8.5%)	90 (28%)	0.39	91 (37%)	73 (30%)	18 (7%)	0.13	0	49 (20%)	0.59	18 (7%)	0.22	24 (10%)	0.042
Male	202 (63%)	39 (12%)	163 (50%)		153 (63%)	108 (44%)	45 (18%)		5 (2%)	86 (35%)		40 (16%)		22 (9%)	
Missing data	6 (2%)	2 (0.6%)	4 (1%)	–	4 (2%)	3 (1%)	1 (0.4%)	–	0	2 (1%)	–	1 (0.4%)	–	1 (0.4%)	–
*Age at admission*															
Median (days)	4	16	3	** < 0.001**	4	6	3	0.03	5	3	–	3	–	16	** < 0.001**
Missing data	13 (4%)	2 (0.6%)	11 (3%)	–	8 (3%)	7 (3%)	1 (0.4%)	–	0	7 (3%)	–	1 (0.4%)	–	0	–
*Stay at NICU*															
Median (days)	5	6	5	0.26	5	7	3	** < 0.001**	5	7	–	3	–	9	** < 0.001**
Missing data	19 (6%)	4 (1%)	15 (5%)	–	10 (4%)	8 (3%)	2 (1%)	–	1 (0.4%)	7 (3%)	–	1 (0.4%)	–	1 (0.4%)	–
*Treatment outcome*															
Deceased	57 (18%)	19 (6%)	38 (12%)	0.012	42 (17%)	35 (14%)	7 (3%)	0.118	1 (0.4%)	22 (9%)	0.69	6 (2%)	0.097	13 (5%)	0.023
Withdrawn	24 (8%)	10 (3%)	14 (4%)	0.1	21 (9%)	17 (7%)	4 (2%)	0.43	0	8 (3%)	0.1	4 (2%)	0.56	9 (4%)	**0.003**
Discharged	34 (11%)	9 (3%)	25 (8%)	0.41	30 (12%)	27 (11%)	3 (1%)	0.3	0	19 (8%)	0.34	3 (2%)	0.051	8 (3%)	0.22
Transferred	204 (64%)	29 (9%)	175 (54%)	** < 0.001**	150 (62%)	100 (40%)	50 (20%)	**0.002**	4 (2%)	85 (34%)	0.54	46 (19%)	**0.003**	15 (6%)	** < 0.001**
Missing data	7 (2%)	2 (0.6%)	5 (2%)	–	5 (2%)	5 (2%)	1 (0.4%)	–	0	3 (1%)	–	0	–	2 (1%)	–
*Diagnosis*															
RSD	203 (65%)	43 (13%)	160 (49%)	0.58	158 (66%)	125 (50%)	33 (13%)	0.018	4 (2%)	95 (38%)	0.076	29 (12%)	**0.008**	30 (12%)	0.69
Pre-term	82 (26%)	17 (5%)	65 (20%)	0.9	70 (29%)	51 (21%)	19 (8%)	0.75	1 (0.4%)	39 (16%)	0.95	17 (7%)	0.88	12 (5%)	0.77
Pneumonia	71 (23%)	21 (6%)	50 (15%)	0.026	58 (24%)	48 (19%)	10 (4%)	0.09	0	31 (13%)	0.65	10 (4%)	0.19	17 (7%)	0.012
Sepsis	41 (13%)	8 (2%)	33 (10%)	0.9	38 (16%)	31 (13%)	7 (3%)	0.26	1 (0.4%)	24 (10%)	0.33	6 (2%)	0.21	7 (3%)	0.98
CHD	26 (8%)	13 (4%)	13 (4%)	** < 0.001**	16 (7%)	13 (5%)	3 (1%)	0.51	1 (0.4%)	5 (2%)	0.039	2 (1%)	0.28	8 (3%)	** < 0.001**
Hyperbilirubinemia	26 (8%)	1 (0.3%)	25 (8%)	0.03	21 (9%)	15 (6%)	6 (2%)	0.76	1 (0.4%)	15 (6%)	0.14	5 (2%)	0.99	0	0.023
PDA	11 (4%)	4 (1%)	7 (2%)	0.18	7 (3%)	5 (2%)	2 (1%)	0.86	0	2 (1%)	0.14	2 (1%)	0.76	3 (1%)	0.087
Gastroenteritis	8 (3%)	1 (0.3%)	7 (2%)	0.58	6 (2%)	4 (2%)	2 (1%)	0.67	0	3 (1%)	0.76	2 (1%)	0.57	1 (0.4%)	0.92
Pulmonary hypertension	7 (2%)	0	7 (2%)	0.18	4 (2%)	1 (0.4%)	3 (1%)	0.023	0	1 (0.4%)	0.21	3 (1%)	0.015	0	0.34
Asphyxia	6 (2%)	1 (0.3%)	5 (2%)	0.83	5 (2%)	4 (2%)	1 (0.4%)	0.77	0	3 (1%)	0.86	1 (0.4%)	0.85	1 (0.4%)	0.92
Pleural effusion	5 (2%)	0	5 (2%)	0.26	5 (2%)	4 (2%)	1 (0.4%)	0.77	0	4 (2%)	0.28	1 (0.4%)	0.85	0	0.29
Other	71 (23%)	18 (6%)	53 (16%)	–	49 (20%)	34 (14%)	15 (6%)	–	1 (0.4%)	23 (9%)	–	14 (6%)	–	11 (4%)	–
Missing data	15 (5%)	6 (2%)	9 (3%)	–	7 (3%)	5 (2%)	2 (1%)	–	0	2 (1%)	–	2 (1%)	–	3 (1%)	–

Since discharge samples were not collected from all patients, patients with admission samples (i.e., all included samples) were analysed as a set in terms of all included patients and characteristics of admission-positive and admission-negative patients, whereas analyses in terms of discharge-positive and discharge-negative were analysed on a smaller set of patients including all patients from whom discharge samples were taken (n = 248). Due to the large number of comparisons, associations were considered significant for *p* < 0.01 (marked in bold). Due to the low number of patients which were positive at admission but negative at discharge (n = 5), this cohort was excluded from the statistical analysis

RSD, Respiratory distress syndrome; CHD, Congenital heart disease; PDA, Persistent ductus arteriosus

Overall, 37% of the patients were female (n = 118). The overall median age of patients in the study was 4 days. Patients CRKP-positive at admission were significantly older compared to patients CRKP-negative at admission (*P* < 0.001) and patients both CRKP-positive at admission and discharge were significantly older (*P* < 0.01) than those CRKP-negative at both admission and discharge and those positive only at discharge. The median length of stay at the NICU overall was 5 days. Patients which were CRKP-positive at discharge had significantly longer NICU stays compared to patients which were CRKP-negative at discharge (*P* < 0.001). Furthermore, a significant difference was observed when comparing both admission and discharge status (*P* < 0.01); patients CRKP-negative at both admission and discharge had shorter NICU stays. Patients transferred from the NICU were found to be significantly likelier to be admission-negative (*P* < 0.001) and discharge-negative (*P* < 0.01). Regarding diagnoses, patients with CRKP-positive faecal samples at either admission or discharge were more likely to be afflicted with respiratory distress syndrome (*P* < 0.01), and patients with congenital heart disease (CHD) were found to be less likely to be admission-positive for CRKP (*P* < 0.001).

### Antimicrobial susceptibility of CRKP isolates from admission and discharge samples

Data on antimicrobial susceptibility is presented in Table 2. Isolates from patients at admission did not significantly differ in antibiotic susceptibility compared to isolates from patients at discharge who were negative at admission (data no shown). None of the admission- or discharge-isolates were susceptible to meropenem (which was an inclusion criterion). Cefotaxime susceptibility rates were also accordingly at 0% for each included isolate. All admission- and discharge-isolates (except one) were MDR. The susceptibility rates for aminoglycosides were low; for gentamicin, amikacin and tobramycin, the susceptibility rates were 41%, 8.8% and 0% for admission-isolates and 36%, 4.3% and 1.1% for discharge-isolates, respectively. Susceptibility ratios were also low for ciprofloxacin and fosfomycin; for admission-isolates, susceptibility ratios were 15% and 5.9% respectively, whereas for discharge-isolates, the corresponding ratios were 6.5% for each antibiotic. Ceftazidime/avibactam susceptibility ratios were also fairly low, 35% of the admission-isolates and 39% of the discharge-isolates were susceptible. The highest susceptibility ratios observed were for tigecycline and colistin. 100% and 96% of the admission- and discharge-isolates were susceptible to tigecycline whereas 82% and 89% were susceptible to colistin, respectively. Notably, for 4 colistin-susceptible admission isolates (12%), heteroresistance was observed (i.e., the main population was susceptible), however, a resistant subpopulation could be isolated from the COL-APSE agar. The colistin-heteroresistant isolates were denoted as resistant in the summary of the antimicrobial susceptibility data.

**Table 2:** Antimicrobial susceptibility testing data for isolates of carbapenem-resistant *Klebsiella pneumoniae* originating from admission, discharge, bloodstream and tracheal aspirate samples

Antibiotic	Admission (n = 34)		Discharge (n = 92)		Discharge (Adm-) (n = 59)		Blood (n = 28)		Tracheal aspirate (n = 13)	
	S	R	S	R	S	R	S	R	S	R
MEM	0	21 (62%)	0	84 (91%)	0	57 (97%)	0	13 (46%)	0	10 (77%)
AK	3 (8.8%)	26 (77%)	4 (4.3%)	76 (83%)	1 (1.7%)	50 (85%)	1 (3.6%)	16 (57%)	0	9 (69%)
CN	14 (41%)	19 (56%)	33 (36%)	47 (51%)	19 (32%)	30 (51%)	5 (18%)	22 (79%)	7 (54%)	4 (31%)
TOB	0	33 (97%)	1 (1.1%)	89 (97%)	0	58 (98%)	0	28 (100%)	0	13 (100%)
CTX	0	34 (100%)	0	92 (100%)	0	59 (100%)	0	28 (100%)	0	13 (100%)
CIP	5 (15%)	28 (82%)	6 (6.5%)	86 (94%)	3 (5.1%)	56 (95%)	3 (11%)	25 (89%)	0	13 (100%)
FOS	2 (5.9%)	32 (94%)	6 (6.5%)	86 (94%)	4 (6.8%)	55 (93%)	3 (11%)	25 (89%)	0	13 (100%)
TGC	34 (100%)	0	88 (96%)	4 (4.3%)	55 (93%)	4 (6.8%)	28 (100%)	0	13 (100%)	0
SXT	17 (50%)	16 (47%)	53 (58%)	38 (41%)	36 (61%)	22 (37%)	17 (61%)	11 (39%)	5 (39%)	8 (62%)
CL	16 (47%)	18 (53%)	40 (44%)	52 (57%)	26 (44%)	33 (56%)	20 (71%)	8 (29%)	4 (31%)	9 (69%)
TE	22 (65%)	12 (35%)	68 (74%)	21 (23%)	48 (81%)	9 (15%)	18 (64%)	10 (36%)	10 (77%)	3 (23%)
CZA	12 (35%)	22 (65%)	36 (39%)	56 (61%)	26 (44%)	33 (56%)	2 (7.1%)	26 (93%)	7 (54%)	6 (46%)
CO	28 (82%)	6 (18%)	82 (89%)	10 (11%)	53 (90%)	6 (10%)	26 (93%)	2 (7.1%)	11 (85%)	2 (15%)

The column denoted “Discharge (Adm-)” contains isolates which are a subset of all the discharge-isolates, the isolates in this column are from patients which had faecal samples at the admission screening which tested negative for carbapenem-resistant *K. pneumoniae*

MEM; meropenem, AK; amikacin, CN; gentamicin, TOB; tobramycin, CTX; cefotaxime, CIP; ciprofloxacin, FOS; fosfomycin, TGC; tigecycline, SXT; trimethoprim-sulfamethoxazole, CL; chloramphenicol, TE; tetracycline, CZA; ceftazidime/avibactam, CO; colistin

### Antibiotic resistance genes among CRKP from admission and discharge samples

**Fig. 1:**
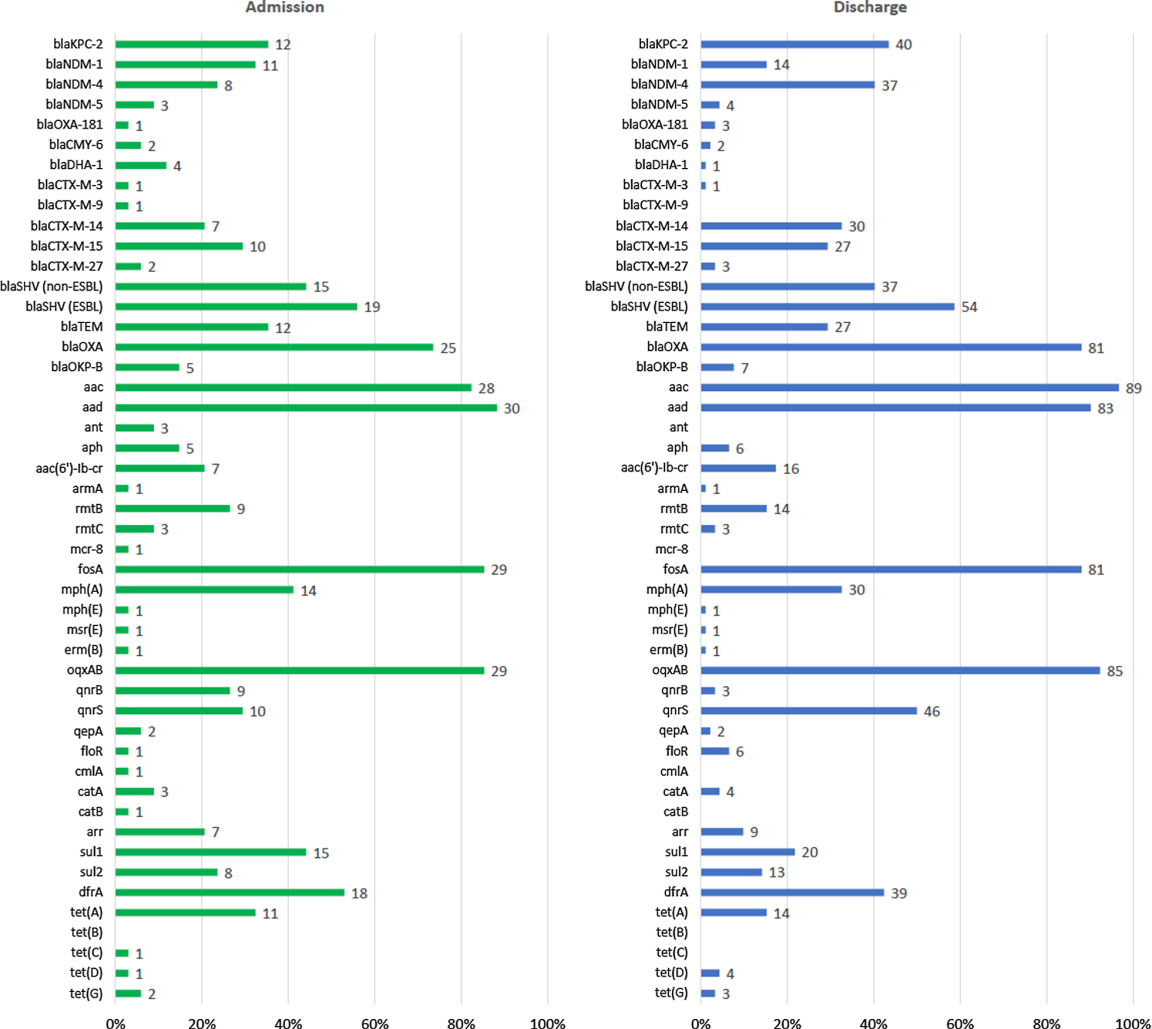
Antibiotic resistance genes detected in isolates of carbapenem-resistant *Klebsiella pneumoniae* originating from faeces samples from patients at admission and discharge, and from clinical isolates from blood and tracheal aspirates. For clarity, certain gene variants (specified below) have been aggregated into gene categories. These are, and consist of: blaSHV (non-ESBL): *bla*_SHV-28_, *bla*_SHV-33_, *bla*_SHV-56_, *bla*_SHV-65_, *bla*_SHV-67_, *bla*_SHV-73_, *bla*_SHV-79_, *bla*_SHV-96_, *bla*_SHV-110_, *bla*_SHV-111_, *bla*_SHV-133_, *bla*_SHV-142_, *bla*_SHV-165_, *bla*_SHV-172_, *bla*_SHV-179_, *bla*_SHV-182_, *bla*_SHV-194_, *bla*_SHV-196_; blaSHV (ESBL): *bla*_SHV-12_, *bla*_SHV-13_, *bla*_SHV-30_, *bla*_SHV-38_, *bla*_SHV-42_, *bla*_SHV-99_, *bla*_SHV-106_; blaTEM: *bla*_TEM-1B_, *bla*_TEM-216_; blaOXA: *bla*_OXA-1_, *bla*_OXA-9_, *bla*_OXA-10_; aac: *aac(3)*-IIa, *aac(3)*-IId, *aac(6’)*-Ib, *aac(6’)*-Ib3, *aac(6’)*-Ib-cr; aad: *aadA1*, *aadA2b*, *aadA3*, *aadA16*, *aadA5*; ant: *ant(2″)*-Ia, *ant(3’)*-VI; aph: *aph(3″)*-Ib, *aph(6)*-Id; blaOKP: *bla*_OKP-B-2_, *bla*_OKP-B-3_, *bla*_OKP-B-4_, *bla*_OKP-B-8_, *bla*_OKP-B-10_, *bla*_OKP-B-14_; fosA: *fosA*, *fosA3*, *fosA5*; oqxAB: *oqxA*, *oqxB*; qnrB: *qnrB1*, *qnrB4*, *qnrB6*, *qnrB9*; catB: *catB3*, *catB8*; arr: *arr-2*, *arr-6*; drfA: *dfrA1*, *dfrA7*, *dfrA12*, *dfrA14*, *dfrA23*, *dfrA27*

Although *bla*_KPC-2_, which was carried by 35% and 44% of the isolates collected at admission and discharge, respectively, was the single most common carbapenemase gene observed among the faecal isolates, genes encoding NDM-type carbapenemases were more common taken together (Fig. 1). For admission-isolates, *bla*_NDM-1_ (32%), *bla*_NDM-4_ (24%) and *bla*_NDM-5_ (8.8%) together constituted 65% if the carbapenemase genes observed whereas for discharge-isolates, the abovementioned NDM-type genes constituted 60% of all carbapenemase genes, with *bla*_NDM-4_ being the most common (40%), followed by *bla*_NDM-1_ (15.2%) and *bla*_NDM-5_ (4.3%). The carbapenemase gene *bla*_OXA-181_ was also observed among a few of the isolates (2.9% of the admission-isolates and 3.3% of the discharge-isolates, respectively). Plasmid-mediated ampC-genes were also detected. Among admission isolates, 4 carried *bla*_DHA-1_ (12%) and 2 carried *bla*_CMY-6_ (5.9%). Among discharge isolates, 1 carried *bla*_DHA-1_ (1.1%) and 2 carried *bla*_CMY-6_ (2.2%)_._ *bla*_CTX-M-14_ and *bla*_CTX-M-15_ were the most common CTX-M-genes detected; their prevalences were, respectively, 21% and 29% among admission isolates, and 33% and 29% for discharge isolates.

A wide range of genes conferring resistance to antibiotics other than β-lactams were observed (Fig. 1). Notably, genes encoding 16S rRNA methylases, conferring high grade aminoglycoside resistance, were carried by 38% of admission-isolates and 20% of discharge-isolates. The most common were *rmtB*, followed by *rmtC* and *armA*, which were carried by 27%, 8.8% and 2.9% of the admission-isolates and 15%, 3.3% and 1.1% of the discharge-isolates, respectively. The colistin resistance gene *mcr-8* was detected in one admission-isolate; the isolate was resistant to colistin and the MIC for the isolate was 16 mg/L.

### Multilocus sequence typing of CRKP isolates from admission and discharge samples

Typing data based on MLST is presented in Table 3. Among both admission- and discharge-isolates, ST15 was found to be the predominant ST. Among the admission-isolates, 14 isolates (41%) were found to belong to ST15. This was followed by ST11, to which 4 isolates (12%) belonged. From the discharge samples, 57 isolates (62%) belonged to ST15. The second most common ST among these isolates was ST14, to which 10 isolates (11%) belonged. This ST was followed in prevalence by ST11 and ST16, to which 7 (7.6%) and 4 isolates (4.3%) belonged, respectively. When the discharge-isolates were stratified on isolates originating from patient with negative admission samples (n = 59), ST15 was found at a significantly higher ratio of 75% (n = 44) (*P* = 0.0015). The second most common ST among these isolates, ST16 was found only among 5 isolates (8.5%). By comparing the STs of isolates from admission samples to isolates from discharge samples originating from the same patient, 19 patients (59%) had faecal isolates belonging to the same ST.

**Table 3:** Sequence types (STs) of isolates of carbapenem-resistant *Klebsiella pneumoniae* originating from admission, discharge, bloodstream and tracheal aspirate samples

ST	Admission	Discharge	Discharge (Adm-)	Blood	Tracheal aspirate
	(n = 34)	(n = 92)	(n = 59)	(n = 28)	(n = 13)
ST11	4 (12%)	7 (7.6%)	2 (3.4%)	1 (3.6%)	0
ST14	1 (2.9%)	10 (11%)	5 (8.5%)	4 (14%)	3 (23%)
ST15	14 (41%)	57 (62%)	44 (75%)	5 (18%)	10 (77%)
ST16	1 (2.9%)	4 (4.3%)	2 (3.4%)	1 (3.6%)	0
ST17	1 (2.9%)	0	0	3 (11%)	0
ST22	2 (5.9%)	1 (1.1%)	0	0	0
ST334	3 (8.8%)	3 (3.2%)	0	2 (7.1%)	0
ST397	0	0	0	4 (14%)	0
ST3003	2 (5.9%)	2 (2.2%)	0	0	0
Others	3 (8.8%)	3 (3.2%)	2 (3.4%)	7 (25%)	0
New Type	3 (8.8%)	4 (4.3%)	1 (1.7%)	0	0
Inconclusive	0	1 (1.1%)	1 (1.7%)	1 (3.6%)	0

The column denoted “Discharge (Adm-)” contains isolates which are a subset of all the discharge-isolates, the isolates in this column are from patients which had faecal samples at the admission screening which tested negative for carbapenem-resistant *K. pneumoniae*. The column denoted “Others” summarizes isolates from all unique STs to which only a single isolate was found to belong. The column denoted “Inconclusive” summarizes isolates for which the whole-genome sequencing data obtained could not be used to conclusively determine the *K. pneumoniae* ST

### Characteristics of CRKP isolated from blood

The 28 bloodstream isolates, collected in 2017 and 2018, showed a predominance of NDM-type carbapenemase genes (Additional file 1: Fig. S1), 26 isolates (93%) carried a *bla*_NDM_-gene among which *bla*_NDM-1_ (43%) and *bla*_NDM-4_ (43%) were the most and equally common, followed by *bla*_NDM-5_ (7.1%). Only two isolates carried other carbapenemase genes, one carried *bla*_KPC-2_ (3.6%) and the other carried *bla*_OXA-181_ (3.6%). The plasmid-mediated ampC-gene *bla*_DHA-1_ was detected among 21.4% of the blood-isolates. *bla*_CTX-M-15_ (50%) and *bla*_CTX-M-14_ (39%) were the most common CTX-M-genes although *bla*_CTX-M-9_ (3.6%) was also detected in 1 isolate. The high-grade aminoglycoside resistance gene *rmtB* was carried by 8 isolates (29%). The susceptibility testing showed that all blood isolates were MDR (Table 2). The aminoglycoside susceptibility among the blood isolates was very low. The susceptibility ratio to gentamicin was 18%, which was the highest for any aminoglycoside. The susceptibility ratio for ceftazidime/avibactam was only 7.1%. Ciprofloxacin and fosfomycin susceptibility ratios were also low, only 3 isolates (11%) were susceptible to each antibiotic. The susceptibility ratio was highest to tigecycline (100%) and colistin (93%), both which were the only antibiotics for which susceptibility ratios were over 90%. Notably, two isolates displayed heteroresistance to colistin as discovered by plating the isolates on COL-APSE agar plates. These isolates were denoted as colistin-resistant in the summary of the data. All blood isolates were susceptible to tigecycline. The MLST analysis of the blood isolates showed that ST15 was the most common ST, to which 5 isolates (18%). This ST was followed in prevalence by ST14 and ST397 (14%, respectively), and ST17 (11%).

### Characteristics of CRKP isolated from tracheal aspirates

Among the isolates from tracheal aspirates collected in 2017 and 2018 from the study hospital, 7 carried *bla*_KPC-2_ (54%) and 6 carried NDM-type carbapenemase-encoding genes (Additional file 1: Fig. S1); 3 carried *bla*_NDM-1_ (23%) and 3 carried *bla*_NDM-4_ (23%). *bla*_CTX-M-15_ (46%) and *bla*_CTX-M-14_ (23%) were the only CTX-M-genes found among these isolates. The susceptibility testing showed that all tracheal aspirate isolates were MDR (Table 2). Although none of the tracheal aspirate isolates were susceptibility to amikacin and tobramycin, 54% were susceptible to gentamicin. None of the isolates were susceptible to ciprofloxacin and fosfomycin. The susceptibility ratio was highest for tigecycline (100%) and colistin (85%). The tracheal aspirate isolates were found to belong to either of two STs, 10 isolates (77%) belonged to ST15 and 3 isolates (23%) belonged to ST14.

### Genetic relatedness of CRKP isolates

**Fig. 2:**
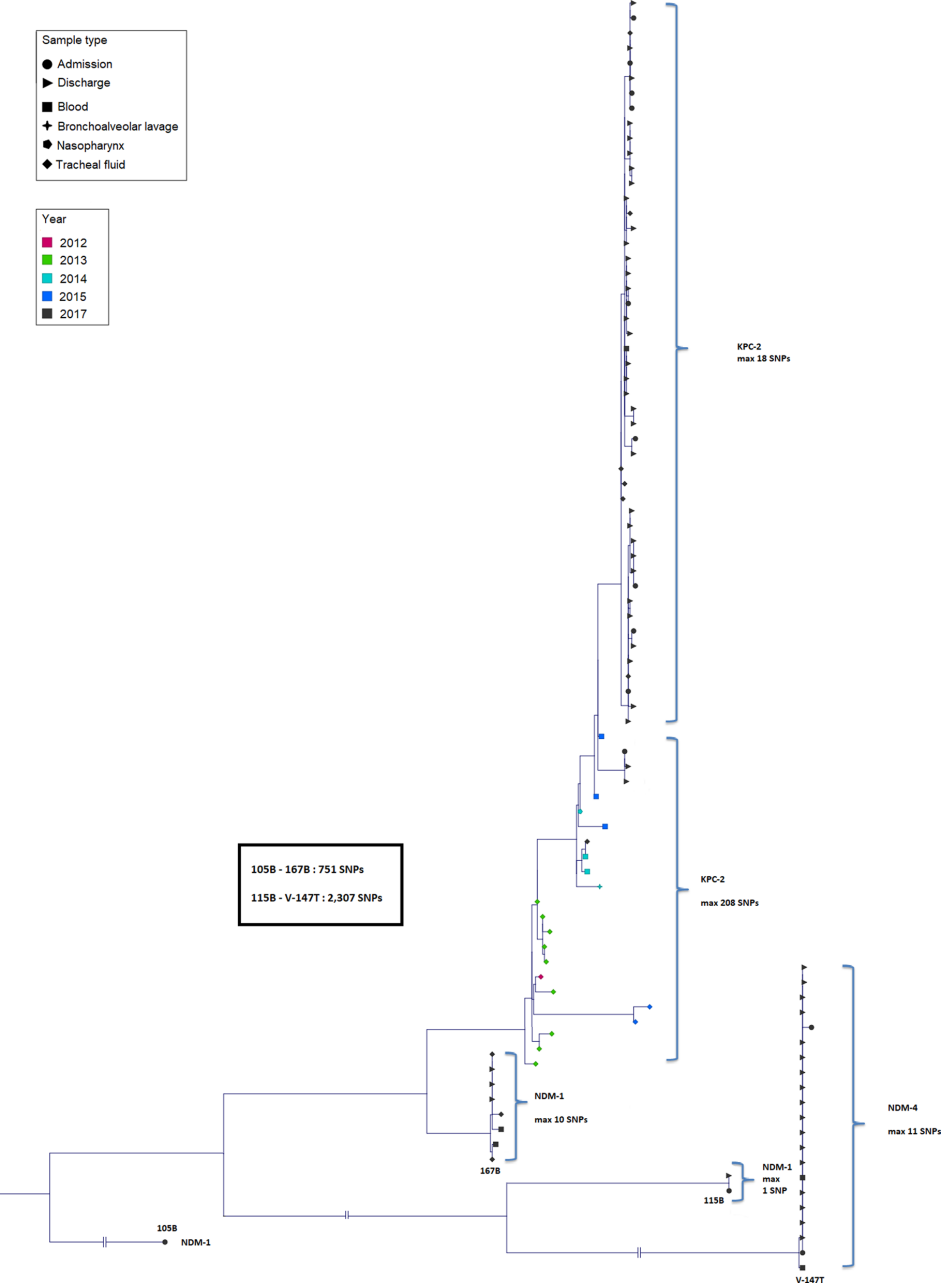
The genetic relatedness based on single-nucleotide polymorphisms (SNPs) of carbapenem-resistant *Klebsiella pneumoniae* belonging to ST15 from the study hospital was illustrated in a phylogenetic tree. Included isolates consisted of 34 isolates from admission samples and 92 isolates from discharge samples collected in 2017 as well as clinical isolates collected from blood, bronchoalveolar lavage, nasopharynx and tracheal fluid from 2015 (n = 5), 2014 (n = 4), 2013 (n = 9) and 2012 (n = 1) at the same hospital. The specimen is denoted by shape and the collection year is denoted by colour. Due to the large difference in SNPs between the clusters, three horizontal axes were truncated (denoted by double pipe symbols). The closest distances between these clusters are denoted by the closest related inter-cluster relatives as indicated in the box. The maximum number of SNPs denoted in the hook parentheses indicate the largest distance between two isolates in the indicated cluster. Also denoted are the carbapenemases encoded by genes carried by the isolates within each cluster

The phylogenetic tree based on the SNP analysis of the isolates belonging to ST15 showed that most isolates clustered in three different clusters (Fig. 2), separated by a minimum distance of > 200 SNPs. Isolates within each cluster differed by ≤ 18 SNPs, indicating that the three clusters comprised three different clones. The largest cluster, consisting of 48 isolates in total, was comprised of 8 admission isolates, 33 discharge isolates, 1 blood isolate and 6 tracheal aspirate isolates. All isolates in this cluster carried *bla*_KPC-2_. Seven of the 8 patients from which the admission isolates originated were also positive for CRKP at discharge. 4 of the patients had discharge isolates which clustered together with the admission isolates. The discharge isolate from 1 patient was also ST15 but differed > 1,800 SNPs from the admission isolate whereas 2 patients carried isolates at discharge which had different STs compared to the admission isolate (ST11 and a novel ST, respectively). The clinical ST15 isolates collected 2012, 2013, 2014 and 2015 formed clusters close to the largest ST15 cluster. The differences between the most closely related ST15 isolates from 2017 and 2015 were 27 SNPs, 2015 and 2014 were 2 SNPs, 2014 and 2013 were 47 SNPs, and 2013 and 2012 were 16 SNPs. The second largest cluster was comprised of 21 isolates, all of which carried *bla*_NDM-4_. 2 isolates in this cluster were recovered from admission samples, 17 from discharge samples and 2 from blood. The third largest cluster consisting of 8 isolates in total, was comprised of 3 discharge isolates, 2 blood isolates and 3 tracheal aspirate isolates.

SNP-analyses were also carried out on isolates belonging to numerous ST11, ST14, ST16 and ST334 isolates (Additional file 2–5: Fig. S2–S5). The ST11-isolates comprised two clusters (maximum distance within clusters were 1 and 2 SNPs, respectively) separated by > 4,000 SNPs. The isolates comprising the larger cluster (n = 9) all carried *bla*_NDM-4_, whereas the 2 isolates comprising the smaller cluster all carried *bla*_NDM-1_. The ST14-isolates (n = 18) formed a single cluster with a ≤ 15 SNPs difference, all of which carried *bla*_NDM-4_. The ST16-isolates (n = 5) clustered together with an outlier isolate differing 45 SNPs from its closest relative. Three isolates carried both *bla*_KPC-2_ and *bla*_NDM-4_ whereas the outlier carried *bla*_OXA-181_. Interestingly, 1 isolate, differing only 2 SNPs from its closest relative, carried *bla*_NDM-1_ and neither *bla*_KPC-2_ nor *bla*_NDM-4_. The ST334-isolates (n = 8) formed a closely related cluster with isolates carrying *bla*_NDM-4_, with an outlier isolate carrying *bla*_NDM-1_ which differed 170 SNPs from its closest relative.

## Discussion

In this study, faecal samples collected at admission and discharge from patients at a NICU in a large Vietnamese hospital were screened for CRKP and the isolates were further characterised with WGS. The results showed a 21% prevalence of CRKP at admission and an increase to 74% at discharge. Statistical analyses revealed several significant associations. Admission-positive patients were significantly older compared with admission-negative patients (*P* < 0.001). Due to the relatively low age of the included patients (median age of all patients was 4 days) this is likely reflecting the longer exposure time of older neonates to the environment. Similarly, significantly longer stays at the NICU (*P* < 0.001) were observed among patients discharge-positive for CRKP, likely reflecting an increased risk by longer exposure time in the NICU environment. Patients transferred from the NICU to other wards of the hospital were found to be significantly likelier to be admission-negative (*P* < 0.001) and discharge-negative (*P* < 0.01) which may reflect more adverse conditions for patients colonized with CRKP, requiring them to stay at the NICU. Respiratory distress syndrome was found to more likely afflict patients who were either positive for CRKP at admission or discharge or both (*P* < 0.01), indicating a possible connection between colonisation and respiratory infection. A significant association was also found that patients with CHD were less likely to be negative for CRKP at admission (*P* < 0.001). Although not significant (*P* = 0.089), patients with CHD tended to be older at admission (median age = 12 days) compared with children without CHD (median age = 4 days), which could explain this association.

A total of 34 isolates from faecal samples collected at admission and 92 collected at discharge were analysed with whole-genome sequencing. MLST and phylogenetic analyses based on SNPs were used to evaluate the phylogenetic relationships between the isolates. ST15 was the most common ST among both admission and discharge isolates. 75% of the discharge isolates from patients which were negative for CRKP at admission belonged to ST15, indicating that the patients colonised during their hospital stay were predominantly colonised with ST15 strains. ST15 is an emerging, global high-risk ST and strains of this ST has been reported on in hospital outbreaks worldwide [16]. Clinical ST15-isolates are often ESBL-producers but have been observed as carbapenemase-producers in several outbreaks. Phylogenetic analysis of the ST15 isolated in the current study showed that most isolates clustered in three different clusters comprised of clones carrying *bla*_KPC-2_, *bla*_NDM-1_ and *bla*_NDM-4_, respectively. To investigate whether the admission- and discharge-isolates were genetically related to clinical isolates, we sequenced and compared blood and tracheal aspirates collected at the study hospital in 2017 and 2018. Each of the clusters consisting of ST15 admission- and discharge-isolates additionally had clinical isolates which clustered together, indicating the potential of the colonising isolates to cause infections. A previous study at the study hospital carried out in 2015 detailed the characteristics of ST15 CRKP which constituted the most common ST among CRKP in clinical specimen [14]. The ST15 from 2015 were found to be comprised of two clones. Representative isolates of these two clones were in the current study shown to be closely related to the ST15 CRKP isolates clustered in the largest cluster among the ST15 isolated in 2017 (all of which carried *bla*_KPC-2_). The closest relatives were separated by only 27 SNPs, indicating that these isolates belong to the same clone, although separated by differences accumulated over two years. Phylogenetic analysis of sporadic isolates of ST15 CRKP carrying *bla*_KPC-2_ recovered in 2012, 2013 and 2014 from clinical specimen at the same hospital further showed a general clustering of the isolates on sampling year, but the close distance between the clusters and the largest cluster of ST15 CRKP from 2017 could indicate clonal descent from the isolate collected in 2012. The two other major clusters of ST15 CRKP (carrying *bla*_NDM-1_ and *bla*_NDM-4_, respectively) from the current study were clearly separated both from each other and the major cluster comprised of isolates carrying *bla*_KPC-2_, indicating the emergence of these two new clones at the hospital. Notably, the phylogenetic analyses also revealed the occurrence of mostly genetically homogenous isolates of other prevalent STs, including ST11, ST14, ST16 and ST334, carrying various carbapenemase resistance genes. These data show that the CRKP at the study hospital are comprised of a diverse set of clones. The data suggests that the ST15 clone has been established and well-adapted to the hospital environment since at least as early as 2015, which may explain its continued predominance in 2017. However, the influx of new clones, including genetically divergent strains of ST15, indicate the need for continued monitoring of potential changes in the epidemiological situation.

All CRKP included in the study were MDR. Resistance ratios to prevalently used antibiotics including aminoglycosides, ciprofloxacin and fosfomycin were high, indicating the difficulty of empirical and culture-based treatment. Notably, a considerable number of isolates carried 16S rRNA methylase genes conferring high grade resistance to aminoglycosides. In our earlier study on isolates collected at the hospital in 2015 [14], we observed a predominance of *bla*_KPC-2_ with a corresponding high ratio of susceptibility to ceftazidime/avibactam. With the emergence of clones carrying *bla*_NDM_-genes, outnumbering *bla*_KPC-2_-carrying isolates in the current data, the susceptibility ratio to ceftazidime/avibactam was considerably lower, reaching as low as 7.1% among blood isolates (Table 2). Colistin is another drug regarded as a last-resort antibiotic for MDR CRKP. The relatively high susceptibility rate observed among isolates in this study (> 80%) suggests colistin’s applicability as such in the current context. Regardless, colistin should be used carefully to avoid selection of resistant clones. Aside from chromosomally mediated colistin resistance (which was the most common mechanism observed), one admission isolate carried the plasmid-mediated colistin resistance gene *mcr-8* and isolates of CRKP with *mcr-1* have previously been reported among clinical isolates at the study hospital [17]. We have previously reported on a high colistin resistance ratio (42%) among the major ST15 clone at the study hospital [18], mainly due to chromosomal alterations, further showing that use of colistin may result in selection of resistant clones and subclones. Tigecycline was the antibiotic to which the highest susceptibility ratio was observed. However, since no clinical breakpoints are available for tigecycline, susceptibility was defined based on an ecological cut-off determined from a wild type population [19]. By using appropriately determined clinical breakpoints, it is possible that the susceptibility would be considered substantially lower. Furthermore, although tigecycline has been used as salvage therapy in critically ill children infected by MDR bacteria, the safety of the antibiotic has not been clearly established in the patient group [20]. Further studies assessing the efficacy and safety of paediatric use of tigecycline are urgently needed.

Notably, no significant difference in antibiotic susceptibility was observed among isolates from patients at admission compared to isolates from patients at discharge which were negative at admission. This likely reflects that CRKP from the study hospital and many CRKP from other hospitals (from which most patients were admitted) share a high degree of genetic homogeneity. In a recent study conducted in four hospitals in Hanoi, CRKP of the same lineages were isolated from three hospitals with patient transfer [21]. However, in the fourth hospital, which did not transfer patients with other hospitals, CRKP were of different lineages, supporting the hypothesis that patient transfer facilitates the dissemination of CRKP strains between hospitals.

In this study, CRKP from faecal samples of patients at admission and discharge at a large Vietnamese NICU were analysed with whole-genome sequencing and most isolates were shown to belong to ST15. Most of these isolates clustered in three different clonal clusters, each also consisting of blood isolates collected at the same hospital. Furthermore, clinical isolates collected from previous years (dating back to 2012) were shown to likely be clonally descended from ST15 isolates in the largest cluster, suggesting a successful hospital strain which can colonise inpatients. The high levels of resistance among the CRKP isolates emphasize the importance of antimicrobial susceptibility testing of clinical isolates of *K.* *pneumoniae* to ascertain an efficient treatment regimen, as the data does not indicate an unequivocal empirical regimen. Aside from prudent usage of antibiotics, infection control measures are also urgently needed to limit the number of HAIs overall. To ensure clinical readiness, continued monitoring of the epidemiological situation is required.

## Supplementary Information

**Additional file 1**: **Fig. S1.** Antibiotic resistance genes detected in isolates of carbapenem-resistant *Klebsiella pneumoniae* originating from clinical isolates collected from blood and tracheal fluid samples. For clarity, certain gene variants (specified below) have been aggregated into gene categories. These are, and consist of: blaSHV (non-ESBL): *bla*_SHV-28_, *bla*_SHV-33_, *bla*_SHV-56_, *bla*_SHV-65_, *bla*_SHV-67_, *bla*_SHV-73_, *bla*_SHV-79_, *bla*_SHV-96_, *bla*_SHV-110_, *bla*_SHV-111_, *bla*_SHV-133_, *bla*_SHV-142_, *bla*_SHV-165_, *bla*_SHV-172_, *bla*_SHV-179_, *bla*_SHV-182_, *bla*_SHV-194_, *bla*_SHV-196_; blaSHV (ESBL): *bla*_SHV-12_, *bla*_SHV-13_, *bla*_SHV-30_, *bla*_SHV-38_, *bla*_SHV-42_, *bla*_SHV-99_, *bla*_SHV-106_; blaTEM: *bla*_TEM-1B_, *bla*_TEM-216_; blaOXA: *bla*_OXA-1_, *bla*_OXA-9_, *bla*_OXA-10_; aac: *aac(3)*-IIa, *aac(3)*-IId, *aac(6’)*-Ib, *aac(6’)*-Ib3, *aac(6’)*-Ib-cr; aad: *aadA1*, *aadA2b*, *aadA3*, *aadA16*, *aadA5*; ant: *ant(2″)*-Ia, *ant(3’)*-VI; aph: *aph(3″)*-Ib, *aph(6)*-Id; blaOKP: *bla*_OKP-B-2_, *bla*_OKP-B-3_, *bla*_OKP-B-4_, *bla*_OKP-B-8_, *bla*_OKP-B-10_, *bla*_OKP-B-14_; fosA: *fosA*, *fosA3*, *fosA5*; oqxAB: *oqxA*, *oqxB*; qnrB: *qnrB1*, *qnrB4*, *qnrB6*, *qnrB9*; catB: *catB3*, *catB8*; arr: *arr-2*, *arr-6*; drfA: *dfrA1*, *dfrA7*, *dfrA12*, *dfrA14*, *dfrA23*, *dfrA27*.

**Additional file 2**: **Fig. S2.** The genetic relatedness based on single-nucleotide polymorphisms (SNPs) of 11 carbapenem-resistant *Klebsiella pneumoniae* (CRKP) belonging to ST11 collected from faecal samples from patients at the study hospital was illustrated in a phylogenetic tree. The closest distances between these clusters are denoted by the closest related inter-cluster relatives as indicated along the horizontal line. The maximum number of SNPs along the clusters indicate the largest distance between two isolates in the indicated cluster.

**Additional file 3**: **Fig. S3.** The genetic relatedness based on single-nucleotide polymorphisms (SNPs) of 18 carbapenem-resistant *Klebsiella pneumoniae* (CRKP) belonging to ST14 collected from faecal samples from patients at the study hospital was illustrated in a phylogenetic tree. The closest distances between these clusters are denoted by the closest related inter-cluster relatives as indicated in parenthesis. The maximum number of SNPs along the clusters indicate the largest distance between two isolates in the indicated cluster.

**Additional file 4**: **Fig. S4.** The genetic relatedness based on single-nucleotide polymorphisms (SNPs) of 5 carbapenem-resistant *Klebsiella pneumoniae* (CRKP) belonging to ST16 collected from faecal samples from patients at the study hospital was illustrated in a phylogenetic tree. The closest distances between these clusters are denoted by the closest related inter-cluster relatives as indicated along the vertical line. The maximum number of SNPs along the clusters indicate the largest distance between two isolates in the indicated cluster.

**Additional file 5**: **Fig. S5.** The genetic relatedness based on single-nucleotide polymorphisms (SNPs) of 8 carbapenem-resistant *Klebsiella pneumoniae* (CRKP) belonging to ST334 collected from faecal samples from patients at the study hospital was illustrated in a phylogenetic tree. The closest distances between these clusters are denoted by the closest related inter-cluster relatives as indicated along the vertical line. The maximum number of SNPs along the clusters indicate the largest distance between two isolates in the indicated cluster.

## Data Availability

All raw reads generated and presented in this study are available at the Sequence Read Archive at NCBI (accession numbers: SRR13256598-SRR13246708).

## Abbreviations

CLSI: Clinical & Laboratory Standards Institute
CRE: Carbapenem-resistant Enterobacteriaceae
CRKP: Carbapenem-resistant *Klebsiella pneumoniae*
ESBL: Extended-spectrum β-lactamase
EUCAST: European Committee on Antimicrobial Susceptibility Testing
HAI: Hospital-acquired infection
ICU: Intensive care unit
LMIC: Low- and middle-income countries
MDR: Multidrug resistance
MLST: Multilocus sequence typing
NICU: Neonatal intensive care unit
SNP: Single-nucleotide polymorphism
WGS: Whole-genome sequencing
