# Supplementary figures and images for: Clonal spread of carbapenem-resistant Klebsiella pneumoniae among patients at admission and discharge at a Vietnamese neonatal intensive care unit
Source: Antimicrob Resist Infect Control. 2021 Nov 20;10:162. doi: 10.1186/s13756-021-01033-3 (PMC8606094; doi:10.1186/s13756-021-01033-3)

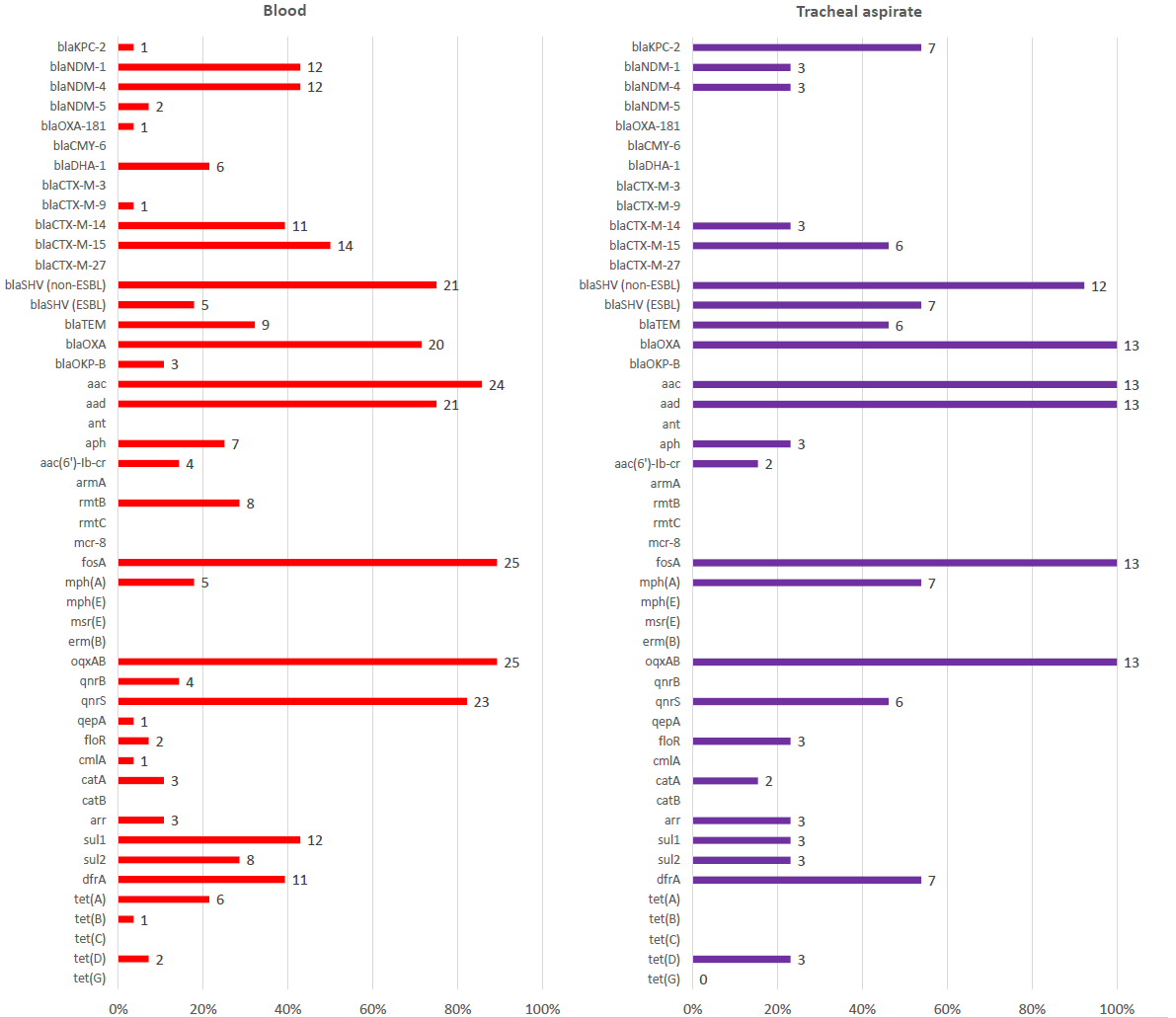

Supplement: Supplementary file 1 — Additional file 1: Fig. S1. Antibiotic resistance genes detected in isolates of carbapenem-resistant Klebsiella pneumoniae originating from clinical isolates collected from blood and tracheal fluid samples. For clarity, certain gene variants (specified below) have been aggregated into gene categories. These are, and consist of: blaSHV (non-ESBL): blaSHV-28, blaSHV-33, blaSHV-56, blaSHV-65, blaSHV-67, blaSHV-73, blaSHV-79, blaSHV-96, blaSHV-110, blaSHV-111, blaSHV-133, blaSHV-142, blaSHV-165, blaSHV-172, blaSHV-179, blaSHV-182, blaSHV-194, blaSHV-196; blaSHV (ESBL): blaSHV-12, blaSHV-13, blaSHV-30, blaSHV-38, blaSHV-42, blaSHV-99, blaSHV-106; blaTEM: blaTEM-1B, blaTEM-216; blaOXA: blaOXA-1, blaOXA-9, blaOXA-10; aac: aac(3)-IIa, aac(3)-IId, aac(6’)-Ib, aac(6’)-Ib3, aac(6’)-Ib-cr; aad: aadA1, aadA2b, aadA3, aadA16, aadA5; ant: ant(2″)-Ia, ant(3’)-VI; aph: aph(3″)-Ib, aph(6)-Id; blaOKP: blaOKP-B-2, blaOKP-B-3, blaOKP-B-4, blaOKP-B-8, blaOKP-B-10, blaOKP-B-14; fosA: fosA, fosA3, fosA5; oqxAB: oqxA, oqxB; qnrB: qnrB1, qnrB4, qnrB6, qnrB9; catB: catB3, catB8; arr: arr-2, arr-6; drfA: dfrA1, dfrA7, dfrA12, dfrA14, dfrA23, dfrA27. [file 13756_2021_1033_MOESM1_ESM.png]

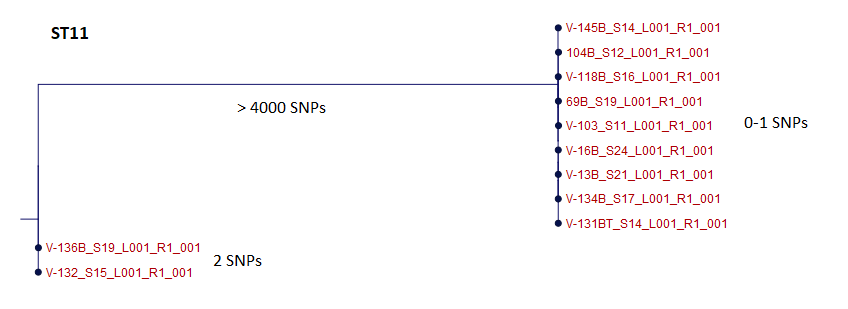

Supplement: Supplementary file 2 — Additional file 2: Fig. S2. The genetic relatedness based on single-nucleotide polymorphisms (SNPs) of 11 carbapenem-resistant Klebsiella pneumoniae (CRKP) belonging to ST11 collected from faecal samples from patients at the study hospital was illustrated in a phylogenetic tree. The closest distances between these clusters are denoted by the closest related inter-cluster relatives as indicated along the horizontal line. The maximum number of SNPs along the clusters indicate the largest distance between two isolates in the indicated cluster. [file 13756_2021_1033_MOESM2_ESM.tif]

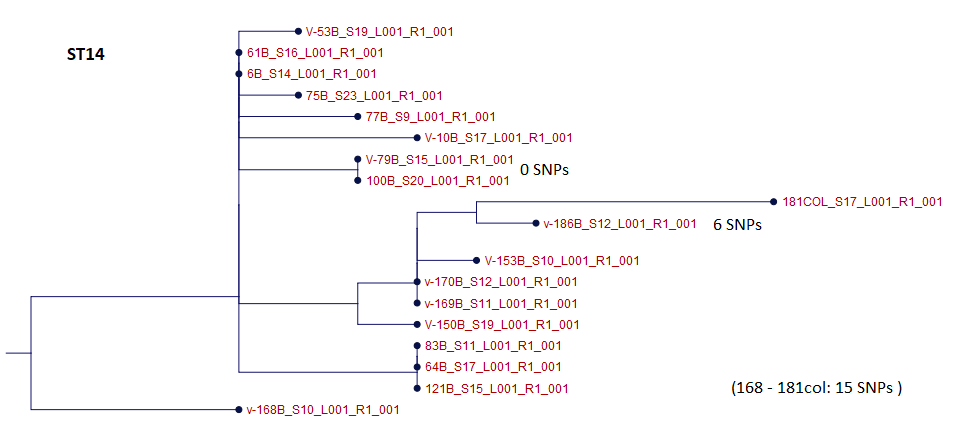

Supplement: Supplementary file 3 — Additional file 3: Fig. S3. The genetic relatedness based on single-nucleotide polymorphisms (SNPs) of 18 carbapenem-resistant Klebsiella pneumoniae (CRKP) belonging to ST14 collected from faecal samples from patients at the study hospital was illustrated in a phylogenetic tree. The closest distances between these clusters are denoted by the closest related inter-cluster relatives as indicated in parenthesis. The maximum number of SNPs along the clusters indicate the largest distance between two isolates in the indicated cluster. [file 13756_2021_1033_MOESM3_ESM.tif]

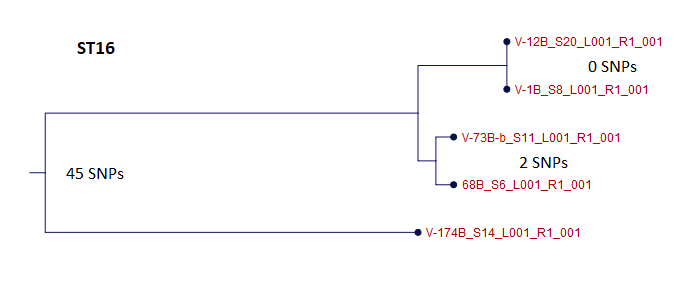

Supplement: Supplementary file 4 — Additional file 4: Fig. S4. The genetic relatedness based on single-nucleotide polymorphisms (SNPs) of 5 carbapenem-resistant Klebsiella pneumoniae (CRKP) belonging to ST16 collected from faecal samples from patients at the study hospital was illustrated in a phylogenetic tree. The closest distances between these clusters are denoted by the closest related inter-cluster relatives as indicated along the vertical line. The maximum number of SNPs along the clusters indicate the largest distance between two isolates in the indicated cluster. [file 13756_2021_1033_MOESM4_ESM.tif]

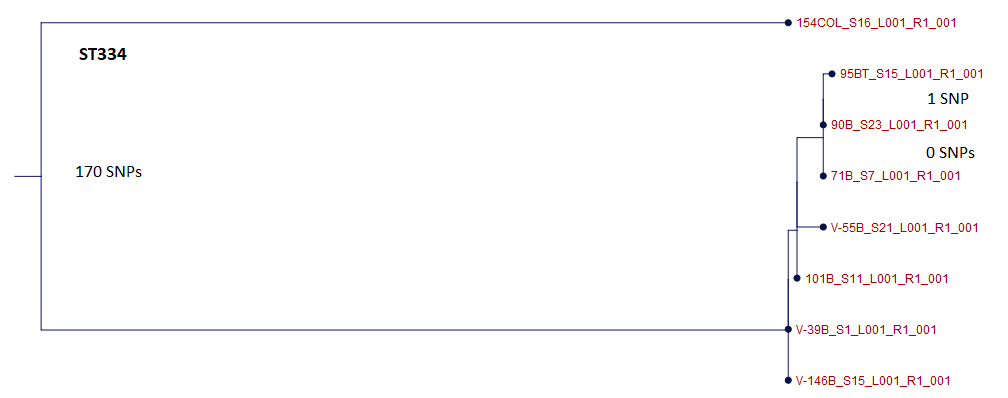

Supplement: Supplementary file 5 — Additional file 5: Fig. S5. The genetic relatedness based on single-nucleotide polymorphisms (SNPs) of 8 carbapenem-resistant Klebsiella pneumoniae (CRKP) belonging to ST334 collected from faecal samples from patients at the study hospital was illustrated in a phylogenetic tree. The closest distances between these clusters are denoted by the closest related inter-cluster relatives as indicated along the vertical line. The maximum number of SNPs along the clusters indicate the largest distance between two isolates in the indicated cluster. [file 13756_2021_1033_MOESM5_ESM.tif]
